# Sequence-specific microscopic visualization of DNA methylation status at satellite repeats in individual cell nuclei and chromosomes

**DOI:** 10.1093/nar/gkt766

**Published:** 2013-08-28

**Authors:** Yufeng Li, Yusuke Miyanari, Kenjiro Shirane, Hirohisa Nitta, Takeo Kubota, Hirofumi Ohashi, Akimitsu Okamoto, Hiroyuki Sasaki

**Affiliations:** ^1^Division of Epigenomics and Development, Medical Institute of Bioregulation, and Epigenome Network Research Center, Kyushu University, Fukuoka 812-8582, Japan, ^2^The Cancer Institute, Tangshan People's Hospital, Hebei 063001, China, ^3^Institut de Génétique et de Biologie Moléculaire et Cellulaire, Université de Strasbourg, F-67404 Illkirch, Cité Universitaire de Strasbourg, France, ^4^Department of Epigenetics Medicine, Interdisciplinary Graduate School of Medicine and Engineering, University of Yamanashi, Yamanashi 409-3898, Japan, ^5^Division of Medical Genetics, Saitama Children’s Medical Center, Saitama 339-8551, Japan and ^6^Research Center for Advanced Science and Technology, The University of Tokyo, Tokyo 113-8656, Japan

## Abstract

Methylation-specific fluorescence *in situ* hybridization (MeFISH) was developed for microscopic visualization of DNA methylation status at specific repeat sequences in individual cells. MeFISH is based on the differential reactivity of 5-methylcytosine and cytosine in target DNA for interstrand complex formation with osmium and bipyridine-containing nucleic acids (ICON). Cell nuclei and chromosomes hybridized with fluorescence-labeled ICON probes for mouse major and minor satellite repeats were treated with osmium for crosslinking. After denaturation, fluorescent signals were retained specifically at satellite repeats in wild-type, but not in DNA methyltransferase triple-knockout (negative control) mouse embryonic stem cells. Moreover, using MeFISH, we successfully detected hypomethylated satellite repeats in cells from patients with immunodeficiency, centromeric instability and facial anomalies syndrome and 5-hydroxymethylated satellite repeats in male germ cells, the latter of which had been considered to be unmethylated based on anti-5-methylcytosine antibody staining. MeFISH will be suitable for a wide range of applications in epigenetics research and medical diagnosis.

## INTRODUCTION

DNA methylation is an important epigenetic modification of the genome in many animals and plants. In mammals, it predominantly occurs at the cytosine base of CpG dinucleotides to produce 5-methylcytosine (5mC). DNA methylation patterns are established and maintained by the members of the DNA methyltransferase family (Dnmt1, Dnmt3a and Dnmt3b) and their associated factors, including Dnmt3L (1). DNA methylation plays crucial roles in the regulation of developmental gene expression, chromatin remodeling, genomic imprinting, X-chromosome inactivation and genome stability (2). Aberrant DNA methylation is an early and fundamental event in the pathogenesis of many human diseases, including cancer (3). Although the mechanism of DNA demethylation has been elusive for decades, recent studies revealed that 5-hydroxymethylcytosine (5hmC) is an important intermediate for replication-dependent and/or replication-independent demethylation (4–6).

A variety of methods have been developed to detect DNA methylation (7). For example, the recent advancement in the high-throughput DNA sequencing technology, along with the use of immunoprecipitation (8), affinity-based pull-down (9) or bisulfite conversion (10), has now made it possible to map 5mC in the genome at base resolution. At the cellular level, global DNA methylation patterns can be microscopically visualized using either anti-5mC antibodies (11,12) or methylated DNA-binding domain fusion proteins (13,14). However, methods for the microscopic visualization of 5mC in specific DNA sequences in individual cells or chromosomes have been lacking. Such an approach may be particularly useful for studying cells that are only available in small numbers, such as early embryonic cells, tissue stem cells, developing germ cells and clinical specimens.

It has been reported that 5mC can be distinguished from cytosine, based on the large difference in osmium oxidation rate (15). Based on this chemistry, a 5mC in target DNA can be detected with a DNA probe containing a bipyridine-attached adenine derivative at the position complementary to the methylatable cytosine when treated with osmium (16). In other words, these interstrand complexes formed by osmium and nucleic acids (ICON) probes allow the sequence-selective detection of 5mC *in vitro* (16). In addition, the ICON probes can also be used to detect 5hmC (17). In this study, we applied this technology to develop a novel method, named methylation-specific fluorescence *in situ* hybridization (MeFISH), for visualizing the DNA methylation status at specific sequences in individual nuclei or chromosomes. MeFISH was able to detect DNA methylation at centromeric and pericentromeric repeat sequences in both mouse and human cells. Notably, a high level of 5hmC at the centromeric repeats was discovered by MeFISH in developing male germ cells. We suggest that this method is suitable for a wide range of applications in epigenetics research.

## MATERIALS AND METHODS

### ICON probes

The ICON probes (Table 1), whose sequences were designed on the basis of the published satellite repeat sequences (18,19), contained a bipyridine-attached adenine derivative at the position corresponding to the methylatable cytosine (Supplementary Figure S1) (16). The probes were synthesized as described (16). In brief, we created a functional nucleoside in which an adenine base and a bipyridine ligand were connected with an alkyl chain (16). The resulting bipyridine-attached nucleoside was then incorporated into a DNA strand by a conventional method using a phosphoamidite form of the nucleoside. For human alpha-satellites and classical satellites 2 and 3, the probes were prepared as locked nucleic acid (LNA)/DNA mixmers, as LNAs provide stable and sensitive FISH probes (20). All probes had an amino group at either the 5′- or 3′-end for labeling (Table 1). The mouse major satellite probe was biotinylated at its 3′ end using biotinamidohexanoyl-6-aminohexanoic acid *N*-hydroxysuccinimide ester (Sigma-Aldrich) for later labeling with fluorescein isothiocyanate (FITC)-labeled avidin (see below). The mouse minor-satellite and human alpha-satellite probes were labeled with Cy3 mono-reactive dye (GE Healthcare). The probe for human classical satellites 2 and 3 was labeled using fluorescein-5-EX succinimidyl ester (Life Technologies). Labeled probes were recovered by ethanol precipitation and further purified by 12.5% denaturing urea polyacrylamide gel electrophoresis.

**Table 1.** ICON probes used for MeFISH

ICON Probe		Specificity	Sequence (5′->3′)	Length (nucleotides)
Mouse	Major satellite	All chromosomes (except Y)	catccacttgacgacttgaaaatgac**B**aaatcactaaaaaacgtg-NH_2_	45
	Minor satellite	All chromosomes (except Y)	cactgttctacaatgcc**B**gttttcaacgtatgtgtttttcagtgtaactc-NH_2_	50
Human	Satellite 2 and 3	Chromosome 1, 9, 16	H_2_N-ggactcGaatgGaataatcatc**B**aatGgaatcGaatgGaatcatc	45
	Alpha satellite	All chromosomes	H_2_N -gctctGtctaaggGaac**B**ttcaactctGtgaGttGaatgcacac	44

Bold capital letter B indicates the bipyridine-modified adenine. Capital letter G in the human probes indicates the LNA nucleotide.

### Preparation of cultured cells and male germ cells

Wild-type (WT, J1) and DNA methyltransferase triple-knockout (*Dnmt*-TKO) mouse embryonic stem (ES) cells (21) cultured under the standard condition on mitotically inactivated mouse embryo fibroblasts (MEFs) were harvested using trypsin–EDTA. MEFs were removed by a cycle of replating. After 30 min of culture, floating cells (ES cells) were collected. A lymphoblast cell line from a healthy female was purchased from the Riken Cell Bank (HEV0190). Lymphoblast cell lines from type 1 (male) and type 2 (female) immunodeficiency, centromeric instability, facial anomalies (ICF) syndrome patients were obtained from a cell bank maintained in the Saitama Children’s Medical Center (22,23). Male germ cells were prepared from mouse testes as described previously (24). Briefly, testes were collected from C57BL/6J fetuses or newborn mice and treated with trypsin–EDTA. Single cells filtered through a cell strainer (40 μm; BD Falcon) were cultured for 3 h and then floating cells (germ cells) were collected.

### Preparation of cell nuclei, chromosome spreads and frozen tissue sections

Cells were washed with phosphate buffered saline (PBS) and pelleted by centrifugation. Cells were gently resuspended in a hypotonic solution (75 mM KCl) and allowed to stand for 10 min at room temperature. Next, the same volume of a methanol–acetic acid fixative solution was added to cells and mixed gently. After centrifugation and removal of the fixative, an equal volume of fresh fixative was added. Additionally, this procedure was repeated twice. After cell nuclei/chromosomes were resuspended in the fixative, a few drops were placed on microscope slides and air-dried. For preparation of fresh frozen sections, fetal testes were embedded in optimal cutting temperature (OCT) compounds and cryosectioned (section thickness, 6 μm). Frozen sections were then fixed in 4% paraformaldehyde.

### MeFISH

A hybridization mixture (4 μl) containing labeled probes (1.25 ng/μl each), 4 × SSC, 0.5 mM EDTA, 10% dextran sulfate and 25% formamide was applied onto slides and then sealed with a coverslip and rubber cement at room temperature. The slides were placed on a heating block at 80°C for 3 min to denature genomic DNA and incubated in a moist chamber at room temperature for at least 2 h. The coverslips were removed by soaking in 2 × SSC, and post-hybridization wash was done three times in 2 × SSC at 37°C. For the biotinylated probe (the mouse major satellite probe), FITC-labeled avidin was added. FISH signals were observed by fluorescence microscopy using appropriate filters. A 15-μl crosslink solution (5 mM K_2_OsO_4_·2H_2_O, 50 mM Tris–HCl, pH 7.7, 0.5 mM EDTA and 1 M NaCl) was added to the slides and incubated in a moist chamber at 37°C for 10 min. We initially included a crosslinking activator, potassium hexacyanoferrate (III) (16), in the osmium solution but later removed it because it was found to produce 5mC-independent background signals even in the absence of osmium (data not shown). Non-crosslinked probe molecules were removed by denaturation in 70% formamide and 2 × SSC at 70°C for 5 min. Finally, the slides were washed in PBS, dehydrated and DNA was counterstained with DAPI in the mounting medium.

### Immunostaining

For immunofluorescence staining of 5mC and 5hmC, cell nuclei/chromosomes fixed on slides were incubated in 4 N HCl at room temperature for 15 min. After neutralization in 100 mM Tris–HCl (pH 8.0) for 10 min, the specimens were incubated with anti-5mC (mouse monoclonal; Calbiochem) and/or anti-5hmC (rabbit polyclonal; Active Motif) antibodies. After blocking with 1% bovine serum albumin and 0.5% Triton X-100 in PBS, the specimens were incubated with the blocking solution containing anti-mouse and/or anti-rabbit secondary antibodies coupled with Alexa Flour 488 or 555 (Life Technologies) at room temperature for 1 h. The samples were then washed and counterstained with DAPI in the mounting medium. For MVH staining, fixed frozen sections were blocked and incubated with an anti-mouse vasa homolog (MVH) antibody (Abcam) at room temperature for 1 h. After several washes, the specimens were incubated with Alexa Flour 488-conjugated secondary antibody, followed by washes and DAPI counterstaining. After observation of the signals, the coverslips were removed by soaking in 2 × SSC, and slides were washed three times in 2 × SSC at 37°C and then subjected to MeFISH.

### Microphotographs and quantification of fluorescence intensity

Fluorescence images were taken under non-saturation conditions using an Olympus Bx51 microscope equipped with an Olympus DP70 digital camera by performing multiple exposures with adequate filters. For comparisons of the FISH and MeFISH images, the stage positions were recorded using the X and Y vernier. The signal intensity was measured by Image-Pro Plus software (Media Cybernetics).

## RESULTS

### Establishment of the MeFISH protocol with mouse ES cells

**Figure 1.**
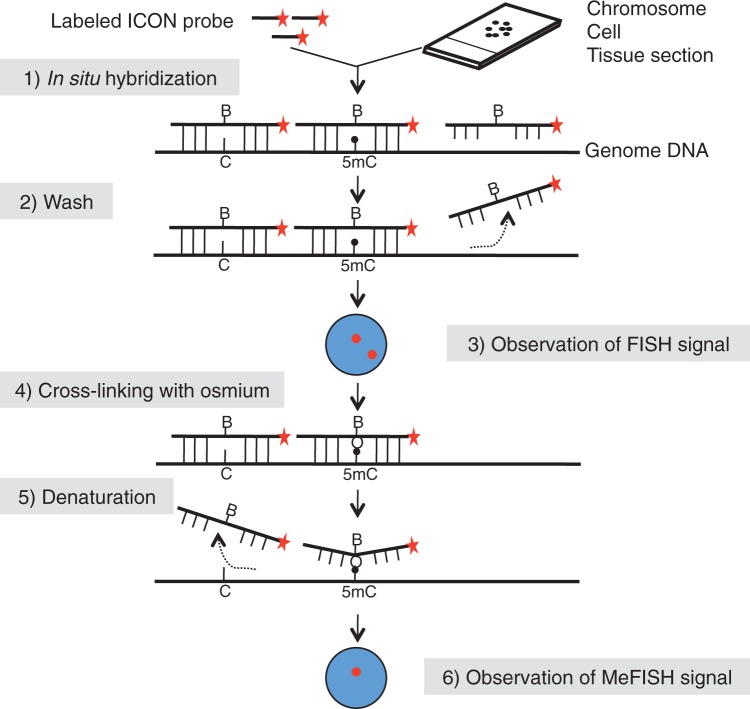
Outline of the MeFISH protocol. Fluorescence-labeled ICON probes were hybridized with heat-denatured cellular DNA on a glass slide. The slide was then washed to remove non-hybridized probes, and FISH signals were observed by fluorescence microscopy. The slide was then treated with osmium for 5mC-dependent crosslinking. After removal of non-crosslinked probes by denaturation, MeFISH signals were observed by fluorescence microscopy. Red stars indicate fluorescence (or biotin) labeling, and red spots in each cell nucleus (blue) represent fluorescence signals. The capital letter ‘B’ in the ICON probe indicates the bipyridine-attached adenine derivative, whereas the capital letters ‘C’ and ‘5mC’ in the target genomic DNA indicate the position of unmethylated and methylated cytosine, respectively.

The overall MeFISH protocol, as outlined in Figure 1, involves conventional FISH technology for sequence-specific target detection and ICON technology for 5mC detection (16). The ICON probes are single-stranded DNA molecules containing a bipyridine-attached adenine at the position complementary to the methylatable cytosine (Supplementary Figure S1) as well as a fluorescent dye (or biotin) at either the 5′- or 3′-end (16). Tissue sections, cells or chromosome spreads are first subjected to FISH with the ICON probes, and the localization of fluorescent signals is then recorded. Next, the specimens are treated with osmium for 5mC-dependent crosslinking, and non-crosslinked probes are removed in a denaturation step. Subsequent comparison of FISH and MeFISH images allows an estimate of the methylation status (see Materials and Methods for details).

To establish the protocol, we used WT and *Dnmt*-TKO mouse ES cells, the latter of which lack all active DNA methyltransferases (*Dnmt1*, *Dnmt3a* and *Dnmt3b*) and have no 5mC (21). We designed ICON probes against major and minor satellite repeats, which are heavily methylated repeat sequences in the pericentromeric and centromeric regions, respectively (25,26). These repeats are present in all chromosomes except Y chromosome (27). The sequences of the ICON probes are shown in Table 1.

**Figure 2.**
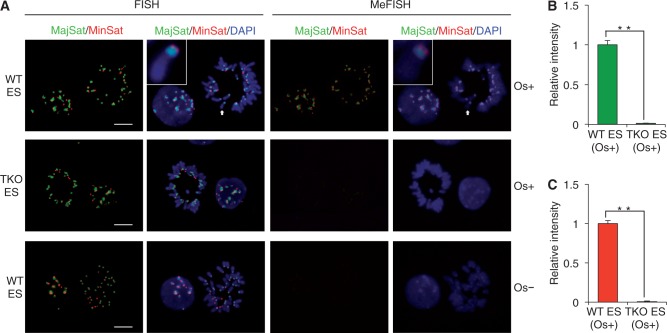
Establishment of MeFISH with mouse ES cells. (**A**) Representative FISH (left) and MeFISH (right) images of mouse major and minor satellites. Cell nuclei and chromosomes prepared from WT and *Dnmt*-TKO mouse ES cells were subjected to two-color MeFISH for major (green) and minor (red) satellites. A preparation from WT ES cells was treated with a crosslink solution without K_2_OsO_4_·2H_2_O (negative control, Os−). Nuclei and chromosomes were counterstained with DAPI (blue). Photographs of different samples were taken under the same exposure conditions. Higher magnification views (insets) of representative chromosomes (indicated by arrow) confirmed the expected signal localization patterns. Bars = 10 μm. (**B** and **C**) Quantification of MeFISH signals of major (B) and minor satellites (C) using Image-Pro Plus. Graphs represent mean ± standard deviation (SD) (*n* = 15, nuclei). The mean intensity obtained from WT ES cells was set as 1. Statistical comparisons are based on the Student's *t*-test. **P < 0.01.

When two-color FISH was performed using these probes, the expected localization patterns were observed (25,26) (Figure 2A, left). On metaphase chromosomes, the major satellite probe displayed a strong signal near the centromeric end, whereas the minor satellite probe often gave a doublet on sister chromatids at the centromeric end. In interphase nuclei, while the major satellite probe gave strong signals that were colocalized with the 4,6-diamidino-2-phenylindole (DAPI)-dense regions, the minor satellite probe gave smaller spots at the periphery of the major satellite signals. The proper localization of the ICON probe signals was also confirmed by co-hybridization with the regular probes (Supplementary Figure S2A and B). The signal intensity was comparable between WT and *Dnmt*-TKO ES cells (Figure 2A, left). The specimens were then treated with osmium and denatured to remove non-crosslinked probes. A reaction time as short as 10 min was enough for 5mC crosslinking at room temperature. Under these conditions (see Materials and Methods), we observed the specific retention of ICON signals in WT but not *Dnmt*-TKO ES cells (Figure 2A, right). Quantitative measurements showed that the MeFISH signals of the major and minor satellites in WT ES cells were much stronger than those in *Dnmt*-TKO ES cells (Figure 2B and C). This strongly suggests that ICON technology is useful for visualizing 5mC of target sequences at both the cellular and chromosomal levels.

### Application of MeFISH to human lymphoblast cells

We applied MeFISH to detect methylation at human satellite repeat sequences. Human classical satellites 2 and 3 and alpha satellites are generally highly methylated; however, they are hypomethylated in cells from patients with ICF syndrome (28). Two types of ICF syndrome have been defined on the basis of their different genetic and epigenetic characteristics. While cases with type 1 ICF have mutations in *DNMT3B* and show normal methylation at alpha satellites, patients with type 2 ICF lack *DNMT3B* mutations and show hypomethylation at alpha satellites (29). Both types of ICF syndrome show hypomethylation at classical satellites 2 and 3 on chromosomes 1, 9 and 16 (29). Recently, mutations in *ZBTB24*, a member of a large transcriptional factor family, were identified in some, but not all, type 2 cases (30–32).

**Figure 3.**
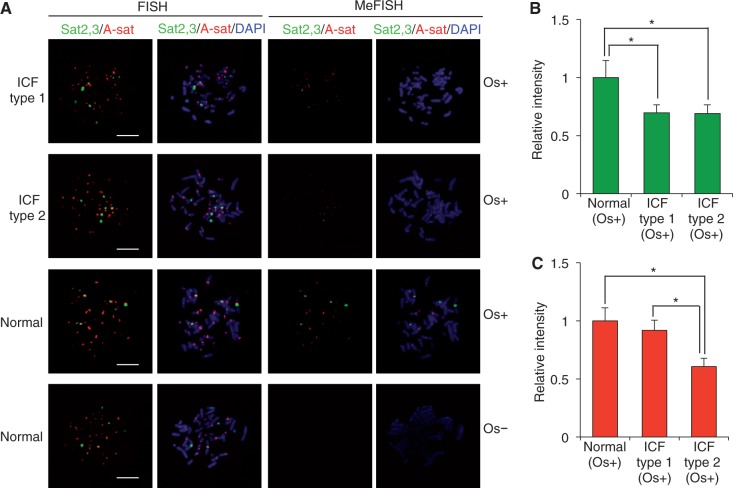
Application of MeFISH to human lymphoblast cells from ICF syndrome. (**A**) Representative FISH (left) and MeFISH (right) images of human classical satellites and alpha satellite. Chromosome spreads prepared from lymphoblast cells of type 1 and type 2 ICF cases and a healthy individual (control) were subjected to two-color MeFISH for classical satellites 2 and 3 (Sat2,3, green) and alpha satellites (A-sat, red). A control preparation was treated with a crosslink solution without osmium (negative control, Os−). Chromosomes were counterstained with DAPI (blue). Photographs of different samples were taken under the same exposure conditions. Bars = 10 μm. (**B** and **C**) Quantification of MeFISH signals of classical satellites 2 and 3 (B) and alpha satellite (C) using Image-Pro Plus. Graphs represent mean ± SD (*n* = 15, nuclei). The mean intensity obtained from control lymphoblast cells was set as 1. Statistical comparisons are based on the Student's *t*-test. *P < 0.05.

Lymphoblast cell lines derived from type 1 and type 2 ICF patients (22,23) were first subjected to Southern blot analysis using methylation-sensitive restriction endonucleases. The methylation patterns at the satellites (Supplementary Figure S3) were consistent with those reported previously (29). We prepared an ICON probe common for classical satellites 2 and 3 (Table 1) because of the high sequence similarity (18). Alpha-satellite FISH signals were observed in the centromeric region of all chromosomes, and classical satellite FISH signals were observed in the pericentromeric region of chromosomes 1 (short arm), 9 (long arm) and 16 (short arm) (Figure 3A, left). The proper localization of the ICON probe signals was also confirmed by co-hybridization with the regular probes (Supplementary Figure S2C and D). When MeFISH signals were observed, the control cells clearly showed both classical- and alpha-satellite signals, whereas weaker signals were detected in type 2 ICF cells under the same photographic exposure conditions (Figure 3A, right). In type 1 ICF cells, although alpha-satellite MeFISH signals were detectable, classical-satellite signals were very weak (Figure 3A, right). Quantitative measurements of the MeFISH signals confirmed the observed differences among the three groups (Figure 3B and C). The MeFISH method was able to reproduce the methylation changes previously reported in type 1 and type 2 ICF cases. Weak MeFISH signals in the ICF specimens are consistent with the fact that these sequences were hypomethylated, but not completely unmethylated, in ICF cells (Supplementary Figure S4) (29,33,34).

### MeFISH detects abundant 5hmC at satellite repeats in male germ cells

In mice, developing male germ cells enter mitotic arrest at around embryonic day 13.5 (E13.5) and resume mitosis after birth. The arrested G1/G0 cells in the fetal testis are called prospermatogonia or gonocytes. During the prospermatogonium stage, many sequences including retrotransposons undergo extensive *de novo* methylation (35). In contrast, major and minor satellites are already substantially methylated in the beginning of the prospermatogonium stage (E13.5) (36,37) and may remain so or become even more highly methylated. However, the centromeric regions in neonatal germ cells showed little staining with anti-5mC antibodies (11). Therefore, it appears that major and minor satellites become demethylated at some point during the late prospermatogonium stage.

**Figure 4.**
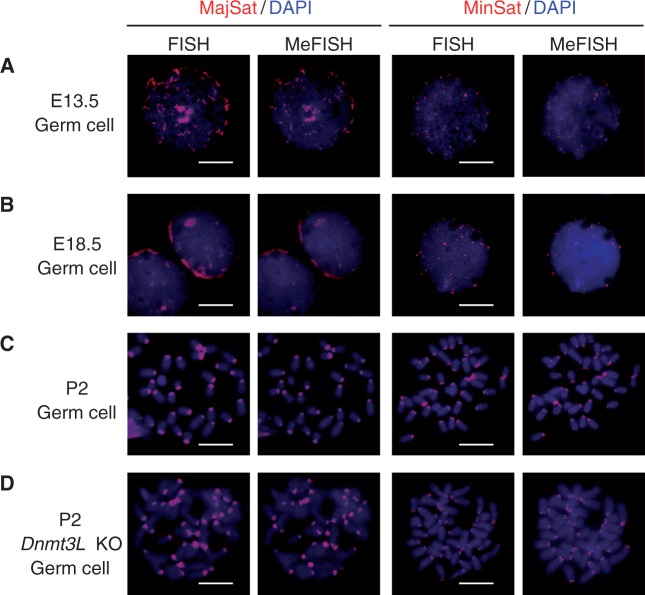
MeFISH in mouse germ cells. Representative FISH and MeFISH images of major and minor satellites in WT male germ cells at embryonic day 13.5 (E13.5) (**A**), E18.5 (**B**) and postnatal day 2 (P2) (**C**) are shown. (**D**) Representative FISH and MeFISH images are also shown for *Dnmt3L*-KO male germ cells at P2. Both satellites show consistent signals (red), with some stage-specific changes in their localization. DNA was stained with DAPI (blue). Bars = 10 μm.

Because the nucleus of prospermatogonia lacks DAPI-dense structure (38), a hallmark of centromeric heterochromatin, MeFISH is advantageous for localizing methylated satellites. The major satellite probe gave focal FISH and MeFISH signals of various sizes in isolated E13.5 prospermatogonia (Figure 4A). The focal signals then moved toward the nuclear periphery and formed stretched clusters in E18.5 prospermatogonia (Figure 4B). The minor satellite probe gave smaller FISH and MeFISH signals in E13.5 prospermatogonia (Figure 4A) and the foci moved toward the nuclear periphery by E18.5 (Figure 4B). The observed localization of these satellites is consistent with that of the centromeres (38). In addition, the results were reproduced in frozen sections of fetal testes, in which prospermatogonia were identified using an anti-MVH antibody (Supplementary Figure S5). These results show that both major and minor satellites are substantially methylated in E13.5 and E18.5 prospermatogonia, further supporting previously published bisulfite data (36,37).

**Figure 5.**
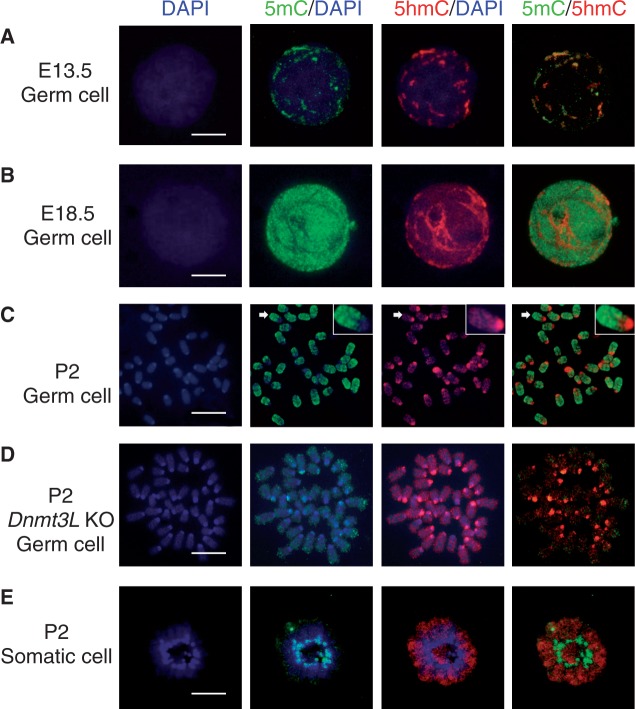
Abundant 5hmC at mouse satellite repeats in fetal and neonatal male germ cells. Immunostaining was done for 5mC and 5hmC in WT mouse male germ cells at E13.5 (**A**), E18.5 (**B**) and P2 (**C**), in *Dnmt3L*-KO germ cells at P2 (**D**) and in somatic cells at P2 (**E)**. Higher magnification views of a representative chromosome (indicated by arrow) are also shown in (C). DNA was stained with DAPI (blue). Bars = 10 μm.

However, we detected substantial MeFISH signals for major and minor satellites on mitotic chromosomes at postnatal day 2 (P2) (Figure 4C), which is in clear contrast to the previous immunostaining data (11). The lack of 5mC staining at the centromeres was confirmed using a commercially available antibody (Figure 5C). As a reference, somatic cells derived from the same specimen showed intense staining at the centromeres (Figure 5E). In addition, the observation of the MeFISH signals in *Dnmt3L*-KO germ cells at P2 (Figure 4D) suggests that the signals are not dependent on *de novo* methylation, since Dnmt3L is essential for *de novo* DNA methylation in prospermatogonia (36).

The conflicting results between MeFISH (Figure 4D) and immunostaining (Figure 5D) may be explained by the fact that osmium reacts with both 5mC and 5hmC (17), whereas anti-5mC antibodies react only with 5mC. 5hmC has been reported in primordial germ cells, pre-implantation embryos, ES cells and brain (4–6,39,40), and is known to be produced by hydroxylation of 5mC, which is catalyzed by ten-eleven translocation (Tet) enzymes. When we examined the chromosomes of P2 germ cells using an anti-5hmC antibody, the centromeres were clearly stained (Figure 5C), strongly supporting our hypothesis. Double staining with anti-5mC and anti-5hmC antibodies showed complementary patterns. Moreover, because the 5hmC signals were observed in *Dnmt3L*-KO germ cells (Figure 5D), we suggest that the formation of 5hmC in P2 germ cells is independent of *de novo* methylation in prospermatogonia. Thus, pre-existing 5mCs were probably the source for the 5hmC production. After the resumption of mitosis at P2, both 5mC and 5hmC signals disappeared in a replication-dependent manner (Supplementary Figure S6), consistent with a previous report (11).

When we examined prospermatogonia in fetal testes at E13.5, the 5hmC signals appeared as discrete foci and colocalized with the 5mC signals (Figure 5A). The localization pattern was similar to that of the satellite MeFISH signals (Figure 4A), suggesting that the MeFISH signals at E13.5 represented both 5mC and 5hmC. At E18.5, the nuclei of prospermatogonia showed a rather uniform 5mC staining (Figure 5B), consistent with the occurrence of global *de novo* methylation, with some less stained ‘streaks’. In these nuclei, the 5hmC signals appeared as elongated clusters (streak-like staining) mainly in the nuclear periphery, coincident with the low-5mC streaks (Figure 5B). The 5hmC staining pattern was similar to that of satellite MeFISH signals (Figure 4B). These results suggest that 5hmCs in the centromeres were produced from pre-existing 5mCs in G1/G0-arrested prospermatogonia.

## DISCUSSION

A new method named MeFISH was developed to visualize DNA methylation at specific target sequences in individual cell nuclei or chromosomes. MeFISH is based on the conventional DNA FISH technology for target detection and the ICON technology for 5mC/5hmC detection. Since we successfully applied this method to five different satellites in humans and mice, we believe that it will be useful for detecting methylation at other satellites, including those in other organisms. We also applied MeFISH to frozen tissue sections and identified cells of interest in these experiments by immunostaining before MeFISH (Supplementary Figure S5).

Our MeFISH results revealed that major and minor satellites at the pericentromeric and centromeric regions are highly 5-hydroxymethylated in male germ cells of neonatal mice (Figures 4 and 5). As the presence of the two cytosine analogues 5mC and 5hmC is mutually exclusive, these results speak in favor of our method: the above-mentioned classes of satellite sequences were previously reported to be hypomethylated using anti-5mC antibodies (11). In addition, we showed that these 5hmCs occur independent of Dnmt3L and are already present in fetal prospermatogonia (Figure 5 and Supplementary Figure S5). We speculate that the production of 5hmCs may involve Tet1 and Tet2, as these enzymes are present in primordial germ cells at E9.5–E10.5 (6). After the resumption of mitosis, both 5hmCs in the centromeres and 5hmCs in the chromosome arms disappeared in a replication-dependent manner (Supplementary Figure S6). The biological significance of this 5hmC enrichment at the centromeres is unknown; however, it is possible that the global demethylation occurring at this stage is somehow linked to the production of spermatogonial stem cells. Male germ cells at later stages are only moderately methylated at major and minor satellites (41).

Several points need to be considered when one conducts MeFISH. First, like other microscopic visualization methods, such as immunostaining, the signal intensity of MeFISH can be affected by the chromatin structure and preparation conditions. Therefore, specimens for comparison should be always included and prepared in parallel. Second, MeFISH signals are inevitably weaker than FISH signals because non-crosslinked probes are removed. This can happen not only on unmethylated targets but also on targets that have a sequence polymorphism at the methylatable cytosine position. The latter possibility is significant because CpG dinucleotides are well-known mutation hotspots and often changed to TpG. Although osmium can react with thymine (42), base pairing between thymine and bipyridine-attached adenine prevents complex formation as long as the modified adenine is located in the middle of the ICON probe (43). Nevertheless, increased signal gain is required to obtain good MeFISH images. Considering these issues, it is always recommended to have appropriate control specimens and/or reference probes.

In conclusion, we have developed MeFISH for sequence-specific detection of 5mC/5mC at satellite repeats. As MeFISH in its current version does not discriminate between 5mC and its analogue 5hmC, we suggest confirmatory immunofluorescence with antibodies specific to the two targets. Therefore, the method is suited for interrogating repeat sequences where we have a priori knowledge of its DNA methylation/hydroxymethylation profile. In principle, the method is applicable to interspersed repeats and single-copy sequences; however, successful applications will need a significantly higher signal/noise ratio, which may be achieved by multiple probes, multiple labeling, signal amplification as well as suppression of non-specific signals.

## ACCESSION NUMBERS

DRA001072.

## SUPPLEMENTARY DATA

Supplementary Data are available at NAR Online, including [44–47].

## FUNDING

Funding for open access charge: Grants-in-Aid for Scientific Research on Priority Area (20062010) from the Ministry of Education, Culture, Sports, Science and Technology of Japan.

*Conflict of interest statement*. None declared.

## Supplementary Material

Supplementary Data
