# Supplementary material for: Sequence-specific microscopic visualization of DNA methylation status at satellite repeats in individual cell nuclei and chromosomes
Source: Nucleic Acids Res. 2013 Aug 28;41(19):e186. doi: 10.1093/nar/gkt766 (PMC3799461; doi:10.1093/nar/gkt766)
Supplement: Supplementary Data [file supp_41_19_e186__index.html]

Sequence-specific microscopic visualization of DNA methylation status at satellite repeats in individual cell nuclei and chromosomes — Sequence-specific microscopic visualization of DNA methylation status at satellite repeats in individual cell nuclei and chromosomes — Supplementary Data 

# Sequence-specific microscopic visualization of DNA methylation status at satellite repeats in individual cell nuclei and chromosomes

## Supplementary Data

files

**Files in this Data Supplement:**

- Supplementary Data - pdf file
