# Supplementary material for: Sequence-specific microscopic visualization of DNA methylation status at satellite repeats in individual cell nuclei and chromosomes
Source: Nucleic Acids Res. 2013 Aug 28;41(19):e186. doi: 10.1093/nar/gkt766 (PMC3799461; doi:10.1093/nar/gkt766)

FIGURE LEGENDS FOR SUPPLEMENTRAY DATA

Supplementary Figure S1 Interstrand cross-linking mediated by an osmium complexation between 5-methylcytosine and a bipyridine-attached adenine derivative. B, bipyridine-attached adenine; M, 5-methylcytosine. This figure is reproduced from reference (16) with permission from American Chemical Society, Washington DC, USA.

Supplementary Figure S2 Proper localization of ICON probe signals confirmed by co-hybridization with regular FISH probes (Supplementary Table S1). Representative images show colocalization of regular FISH probe signals (green) and ICON probe signals (red) for mouse major satellite (**A**), mouse minor satellite (**B**), human classical satellites 2 and 3 (**C**) and human alpha-satellite (**D**). Chromosomes were prepared from WT mouse ES cells and normal human lymphoblast cells. DNA was stained with DAPI (blue). Bars=10 μm.

Supplementary Figure S3 Confirmation of the methylation statuses of classical satellite 2 and alpha satellites in different types of ICF syndrome. Genomic DNA from the same lymphoblast cells described in Figure 3 was subjected to the Southern blot analysis with methylation-sensitive restriction endonucleases *Bst*B1 (classical satellite 2) and *Hha*I (alpha satellite). Lanes 1, type 1 ICF; 2, type 2 ICF; N, normal control lymphoblast cells. Hybridization probes were prepared as described (18). Note that classical satellite 2 was hypomethylated in both ICF types, whereas alpha satellite was hypomethylated only in type 2.

Supplementary Figure S4 Methylome analysis of lymphoblast cells from a healthy donor, a type 1 ICF patient and a type 2 ICF patient. Methylation levels of satellite 2 and alpha satellite were calculated using the data from whole genome bisulfite shotgun sequencing. More than 150 million reads were obtained from each sample and those uniquely mapped to satellite 2 and alpha satellite were used. A maximum of two mismatches were allowed. The numbers of CpGs used to calculate the methylation levels were 123,353 (satellite 2) and 859,255 (alpha satellite) for control; 105,853 (satellite 2) and 582,893 (alpha satellite) for type 1 ICF; 178,501 (satellite 2) and 828,532 (alpha satellite) for type 2 ICF. The bisulfite conversion rate was estimated to be 99.5% for all experiments based on the data from unmethylated lambda phage DNA spiked in the samples. The methylation levels are relatively well correlated with the signal quantification data in Figure 3B and C.

Supplementary Figure S5 Representative FISH and MeFISH images for major and minor satellites in frozen sections of mouse fetal testes. Germ cells were identified by immunostaining for MVH (green). DNA was stained with DAPI (blue). Dashed lines indicate the outlines of seminiferous tubules. Bars=10 μm.

Supplementary Figure S6 Replication-dependent loss of 5mC and 5hmC in spermatogonial chromosomes after the resumption of mitosis. Chromosome spreads were prepared from mouse spermatogonia collected at P2-P4. DNA was stained with DAPI (blue). Immunofluorescence costaining was performed with 5mC (green) and 5hmC antibodies (red). Bars=10 μm.

SUPPLEMENTARY METHODS

Methylome analysis

Genomic DNA of 100 ng spiked with 1 ng of unmethylated lambda phage DNA (Promega) was used for bisulfite treatment using MethylCode Bisulfite Conversion Kit (Invitrogen). Whole genome bisulfite shotgun libraries were prepared using the post-bisulfite adaptor tagging (PBAT) method (43). Concentrations of the PBAT libraries were determined by quantitative PCR (qPCR) using Library Quantification Kit - Illumina (Kapa Biosystems). All sequencing runs were single-ended and 101 nucleotides (nt) in length, and performed on Illumina HiSeq 2500 platform. Based on the qPCR, 13 pM of the PBAT libraries was sequenced per lane. Cluster generation and sequencing were performed in single-read mode using the TruSeq SR Cluster Kit v3-cBot-HS (Illumina) and the TruSeq SBS Kit v3-HS (Illumina). Sequenced reads were processed using the standard Illumina base caller (v.1.8.2). We truncated raw reads to 96 nt to remove any remaining adapter sequences incorporated in the read and lower quality bases near the end of the reads using NGS QC Toolkit (44). The resulting reads were aligned to the reference genome (human hg19) using Bismark alignment software v.0.7.12 (45) with a maximum of two mismatches, and only uniquely aligned reads were retained. We estimated bisulfite conversion rates using reads uniquely aligned to the lambda phage genome. Repeat sequences for hg19 genome were downloaded from UCSC Genome Browser (46).

SUPPLEMENTARY REFERENCES

16. Tanaka, K., Tainaka, K., Umemoto, T., Nomura, A. and Okamoto, A. (2007) An osmium-DNA interstrand complex: application to facile DNA methylation analysis. *J. Am. Chem. Soc.*, **129**, 14511-14517.
18. Tagarro, I., Fernandez-Peralta, A.M. and Gonzalez-Aguilera, J.J. (1994) Chromosomal localization of human satellites 2 and 3 by a FISH method using oligonucleotides as probes. *Hum. Genet.*, **93**, 383-388.
19. Lehnertz, B., Ueda, Y., Derijck, A.A., Braunschweig, U., Perez-Burgos, L., Kubicek, S., Chen, T., Li, E., Jenuwein, T. and Peters, A.H. (2003) Suv39h-mediated histone H3 lysine 9 methylation directs DNA methylation to major satellite repeats at pericentric heterochromatin. *Curr. Biol.*, **13**, 1192-1200.
43. Miura, F., Enomoto, Y., Dairiki, R. and Ito, T. (2012) Amplification-free whole-genome bisulfite sequencing by post-bisulfite adaptor tagging. *Nucleic Acids Res.*, **40**, e136.
44. Patel, R.K. and Jain, M. (2012) NGS QC Toolkit: a toolkit for quality control of next generation sequencing data. *PLoS One*, **7**, e30619.
45. Krueger, F. and Andrews, S.R. (2011) Bismark: a flexible aligner and methylation caller for Bisulfite-Seq applications. *Bioinformatics*, **27**, 1571-1572.
46. Meyer, L.R., Zweig, A.S., Hinrichs, A.S., Karolchik, D., Kuhn, R.M., Wong, M., Sloan, C.A., Rosenbloom, K.R., Roe, G., Rhead, B. *et al.* (2013) The UCSC Genome Browser database: extensions and updates 2013. *Nucleic Acids Res.*, **41**, D64-69.

Supplementary Table 1. Regular FISH probes without bipyridine-attached adenine

Probe		Specificity	Sequence (5'->3')	Length (nucleotides)	reference
Mouse	Major satellite	All chromosomes (except Y)	FITC- ctgtaggacgtggaatatggcaaga	25	Lehnertz *et al*. 2003
	Minor satellite	All chromosomes (except Y)	FITC- gataaaaaccacactgtagaacata	25	Lehnertz *et al*. 2003
Human	Satellite 2 and 3	Chromosomes 1, 9, 16	FITC- tcgagtccattcgatgat	18	Tagarro *et al*. 1994
	Alpha satellite	All chromosomes	FITC- ccaccataggcctcaaagcgctcca	25	GenBank: S67971.1

Yufeng Li_Supplementary Figure S1

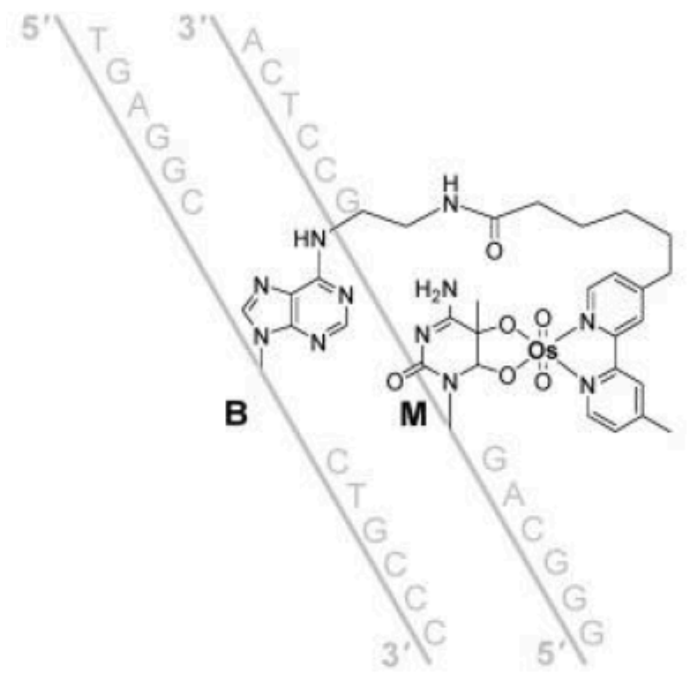

Yufeng Li_Supplementary Figure S2

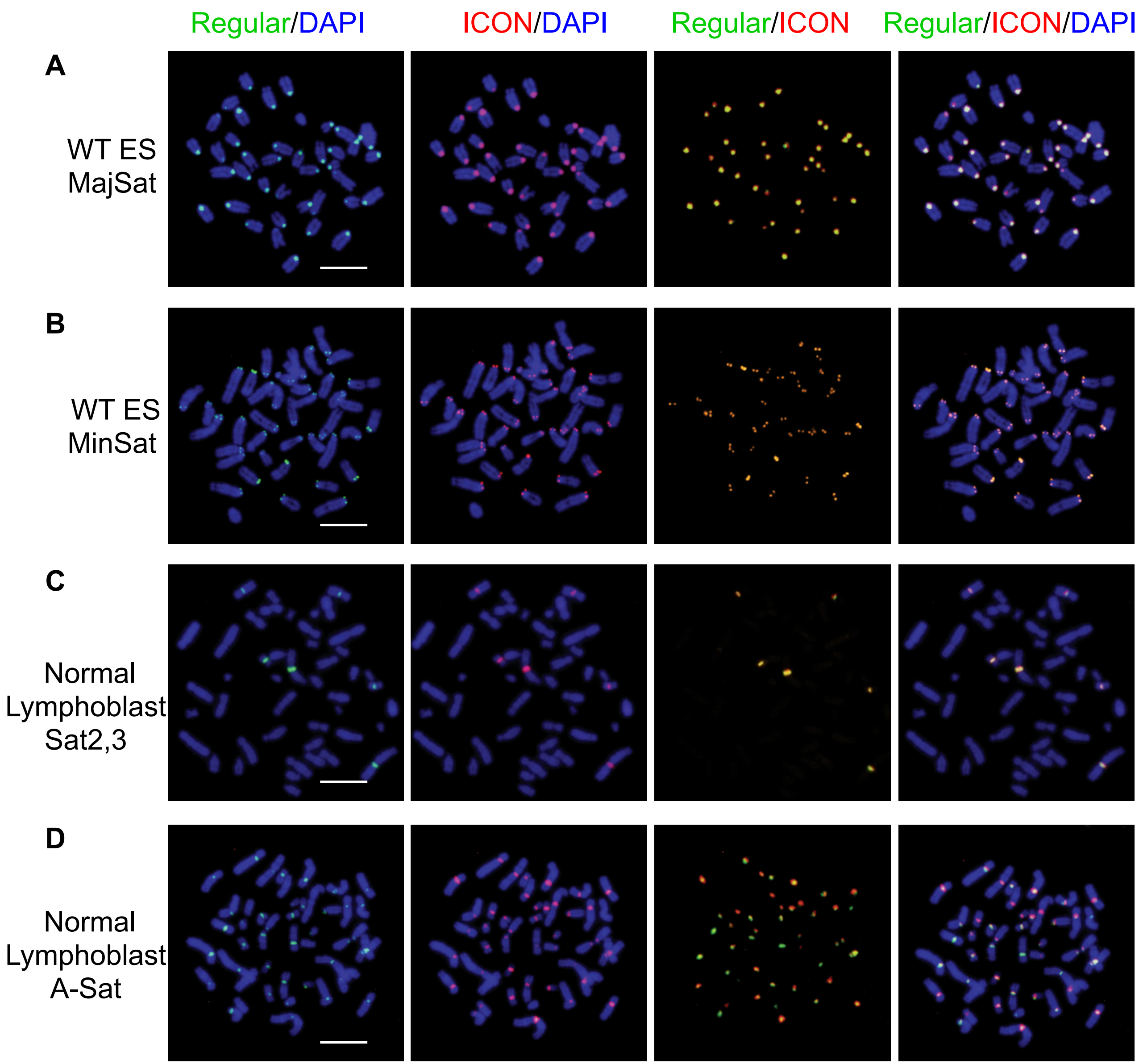

Yufeng Li_Supplementary Figure S3

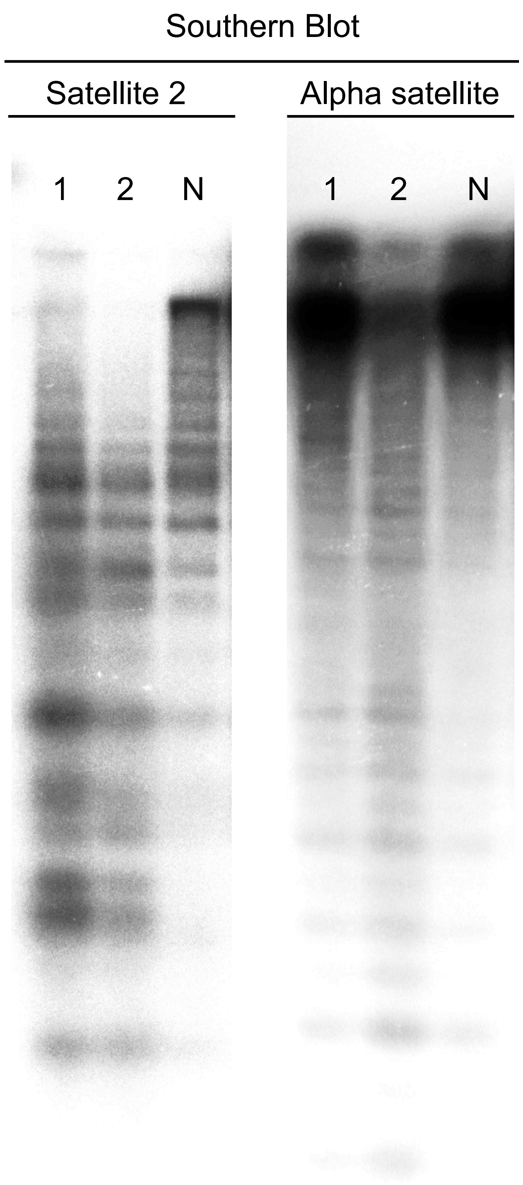

Yufeng Li_Supplementary Figure S4

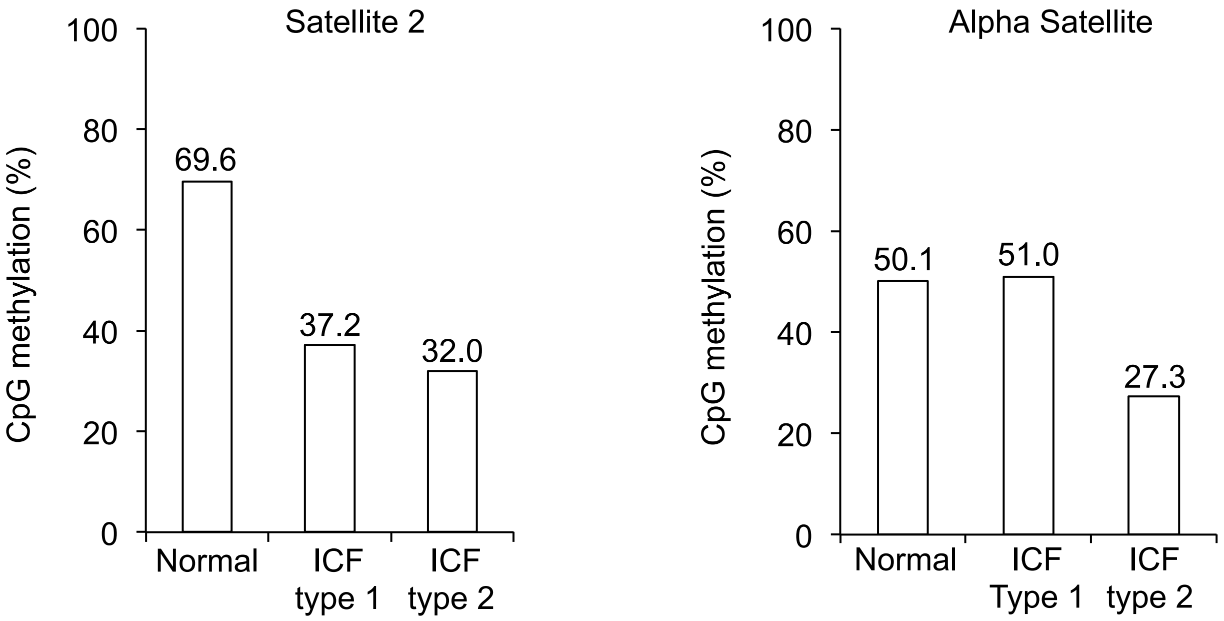

Yufeng Li_Supplementary Figure S5

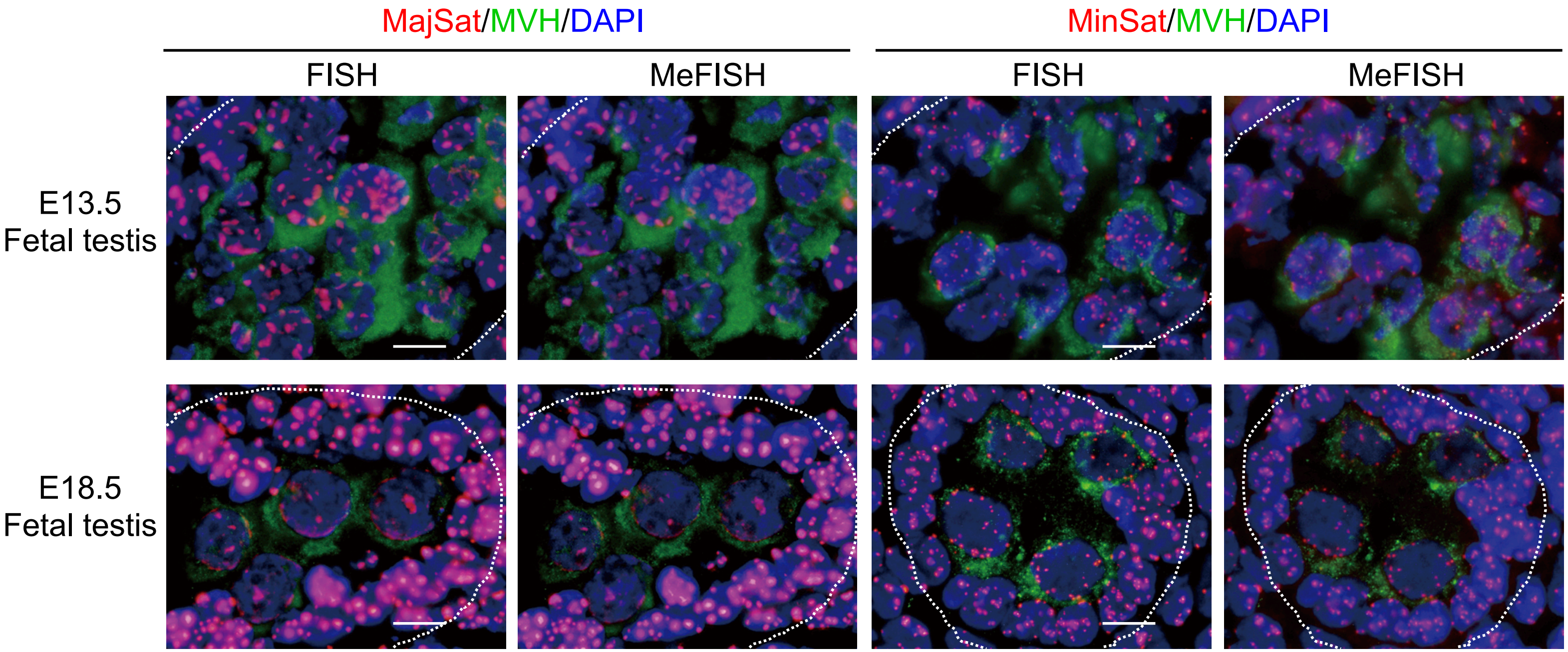

Yufeng Li_Supplementary Figure S6

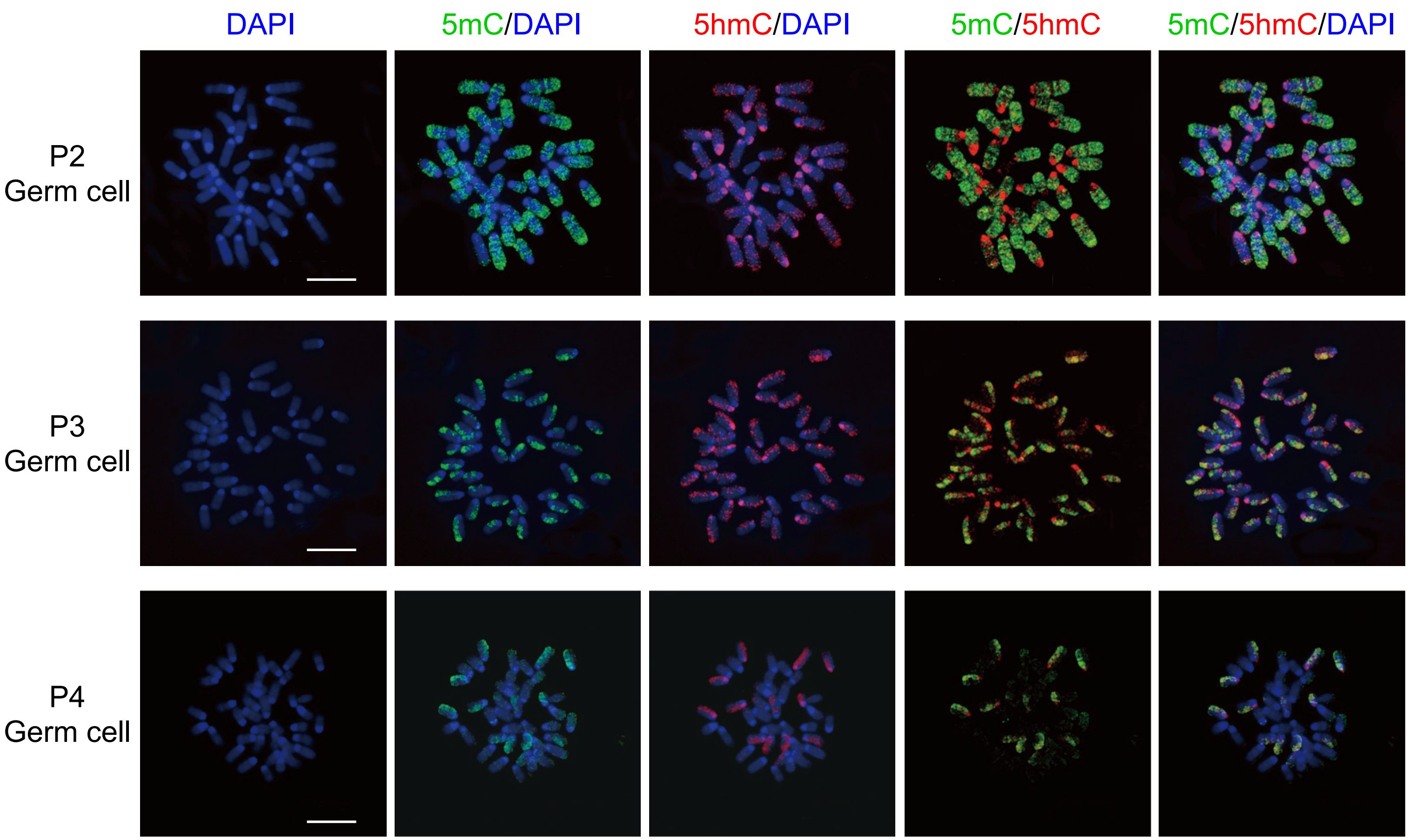

Supplement: Supplementary Data [file supp_gkt766_nar-01038-met-k-2013-File002.pdf]
